# RP1-59D14.5 triggers autophagy and represses tumorigenesis and progression of prostate cancer via activation of the Hippo signaling pathway

**DOI:** 10.1038/s41419-022-04865-y

**Published:** 2022-05-13

**Authors:** Bing Zhong, Zexue Zhao, Xi Jiang

**Affiliations:** 1 Department of Urology, the Affiliated Huai’an No.1 People’s Hospital of Nanjing Medical University, Huai’an, Jiangsu China; 2 Department of Orthopedics, the Affiliated Huai’an No.1 People’s Hospital of Nanjing Medical University, Huai’an, Jiangsu China

**Keywords:** Cancer, Cell biology

## Abstract

Prostate cancer (PCa) is one of the major malignant tumors among men worldwide. Long noncoding RNAs (lncRNAs) have been documented as important modulators in human cancers, including PCa. In our study, we investigated the role and potential mechanism of RP1-59D14.5 in PCa. RP1-59D14.5 expressed at a low level in PCa cells. Gain-of-function assays including colony formation and transwell assays displayed that RP1-59D14.5 overexpression repressed PCa cell proliferation, migration, and invasion. Besides, RP1-59D14.5 up-regulation induced autophagy in PCa cells. Mechanically, luciferase reporter assays and western blot verified that RP1-59D14.5 activated the Hippo pathway in PCa cells. Through RNA-binding protein immunoprecipitation (RIP) and RNA pull-down assays, we validated that RP1-59D14.5 functioned as a competing endogenous RNA (ceRNA) to regulate large tumor suppressor kinase 1/2 (LATS1/2) via targeting miR-147a. Moreover, RP1-59D14.5 recruited HUR to promote casein kinase 1 (CK1) expression. Collectively, RP1-59D14.5 promoted yes-associated protein (YAP) degradation to activate the Hippo pathway in PCa progression via targeting the miR-147a/LATS1/2 axis and recruiting HUR to promote the interaction of CK1 and β-transducin repeat-containing protein (βTrCP). These results implied that RP1-59D14.5 acted as a tumor suppressor in PCa, which might be a target for PCa treatment.

## Introduction

Prostate cancer (PCa) is identified to be the second main cause of cancer-related death among men in the USA [1]. Most of PCa patients at early stage are treated with radical prostatectomy and androgen deprivation therapy [2]. In recent years, despite the development of the economy and the improvement of therapeutic methods, the treatment outcome for patients with advanced PCa is still unsatisfactory [3, 4]. Hence, further understanding of molecular mechanisms underlying PCa progression is greatly important for identifying effective targets for PCa treatment.

Non-coding RNAs such as lncRNAs, microRNAs (miRNAs), and pseudogenes were initially considered “junk genes”. However, their roles in the modulation of human cancers have been brought into focus now [5]. LncRNAs are implicated in a variety of biological processes, such as cell growth, cell proliferation, and cell apoptosis [6]. Increasing evidence has indicated that lncRNA can be used as a diagnostic and prognostic target for cancers [7]. It has been widely documented that lncRNAs can play an oncogenic or tumor suppressive role in PCa [8, 9]. HCG11 suppresses PI3K/AKT signaling pathway to impede PCa progression via targeting miR-543 [10]. CASC15 targets miR-200a-3p to enhance cell migration and invasion in PCa [11]. Functionally, lncRNAs may impact the function of transcriptional complexes, regulate chromatin structures via acting as scaffolds between proteins, or serve as a ceRNA to target miRNAs [12, 13]. However, there are still some unidentified lncRNAs in PCa.

Autophagy is a process in which subcellular membranes are subjected to dynamic morphological changes which result in the degradation of cellular proteins and cytoplasmic organelles [14]. Autophagy is a significant cellular response to stress or starvation. Recent studies have suggested that autophagy plays a crucial role in human cancers, including PCa [15]. However, the studies on lncRNA and autophagy in PCa remain poorly reported.

Hippo signaling pathway is evolutionarily conserved and plays a critical role in the tumorigenesis and development of human cancer [16]. YAP and transcriptional coactivator with PDZ-binding motif (TAZ) are the main effectors of the Hippo pathway, and they are associated with various transcription factors such as transcriptional enhanced associate domains (TEADs), inducing a growth-promoting gene expression to affect cell proliferation, apoptosis, and migration [17]. Meanwhile, YAP may be inactivated through its phosphorylation by upstream kinases LATS1/2 [18]. However, the interaction between lncRNA and the Hippo pathway in PCa remains to be further investigated.

In our study, we investigated the function of RP1-59D14.5 in PCa. We detected RP1-59D14.5 expression in PCa and determined the impacts of RP1-59D14.5 on cell proliferation, migration, invasion, and autophagy in PCa. Moreover, we analyzed the molecular mechanism underlying the interaction between RP1-59D14.5 and Hippo pathway in PCa cells. In conclusion, our study demonstrated the important role of RP1-59D14.5 in PCa, which might shed light on PCa treatment.

## Materials and methods

### Cell culture and treatment

Human PCa cell lines (LNCaP, PC3, and DU145) and human normal prostatic epithelial cell lines (RWPE-1) were purchased from ATCC (Manassas, VA, USA). LNCaP was grown in RPMI-1640 medium (Invitrogen, Carlsbad, CA, USA). PC3 was grown in F-12K medium (Invitrogen). DU145 was grown in Eagle’s Minimum Essential Medium (Invitrogen). RWPE-1 was grown in Keratinocyte Serum Free Medium (K-SFM; Invitrogen). In total 10% fetal bovine serum (FBS) and 1% penicillin-streptomycin were both procured from Gibco (Grand Island, NY, USA) and used to supplement cell culture medium. Cell culture was achieved in 5% CO_2_ at 37 °C.

### Cell transfection

For cell transfection, cells were seeded into 6-well plates. For overexpression of RP1-59D14.5 and HUR, pcDNA3.1 vectors (GeneChem, Shanghai, China) were subcloned with RP1-59D14.5 and HUR (pcDNA3.1/RP1-59D14.5 and pcDNA3.1/HUR). Empty pcDNA3.1 vector was served as negative control. Short hairpin RNA (shRNA) against CK1 (sh/CK1) was designed and synthesized by GenePharma (Shanghai, China). To overexpress miR-147a, miR-147a mimics (GenePharma) was transfected into PCa cells, using NC mimics as control. Lipofectamine 3000 (Invitrogen) was applied for 48 h transfection.

### Quantitative real-time polymerase chain reaction (RT-qPCR)

Extraction of total RNA from cells was conducted using TRIzol reagent (Thermo Fisher Scientific, IL, USA). RNA was reversely transcribed into cDNA using Reverse Transcription Kit (Toyobo, Osaka, Japan). To evaluate gene expression, qPCR was conducted using SYBR Green PCR Master Mix (Takara, Kyoto, Japan). Relative expression was normalized to GAPDH or U6 using 2^−ΔΔCt^ method. The experiment was conducted at least three times.

### Colony formation

Cells (600 cells per well) were planted into 6-well plates and cultured for 14 days. Colonies were washed with PBS, fixed with 4% paraformaldehyde and then stained with 0.5% crystal violet. Finally, the number of colonies was counted manually. The experiment was conducted at least three times.

### 5-ethynyl-2′-deoxyuridine (EdU)

EdU assay was performed in cells using EdU Cell Proliferation Kit (Beyotime, Shanghai, China) with Alexa Fluor 594. Cells were treated with EdU medium diluent and DAPI staining solution, and observed using an inverted microscope (Olympus, Tokyo, Japan). The experiment was conducted at least three times.

### Transwell assays

Cell invasion and migration assays were conducted using Transwell chambers with or without Matrigel (BD, Biosciences). Cells were seeded on Transwell upper chambers with or without Matrigel. Serum-free medium was added to the upper chambers. Medium containing 20% FBS was placed in the lower chamber. The migratory or invasive cells attached to the lower chamber were fixed with 4% paraformaldehyde and stained with 0.5% crystal violet. Then, cells were photographed under a microscope. Experiments were repeated at least three times.

### Transmission electron microscope (TEM)

Cells (2 × 10^7^ cells per well) were centrifuged and fixed with glutaraldehyde solution for 12 h. Then, cells were added with osmic acid solution for 2 h at 4 °C. Next, cells were embedded with epoxy resin and sliced into sections (100 nm thick). Finally, uranyl propionate and lead citrate were used for staining and the sections were observed under a transmission electron microscope and photographed. The experiment was conducted at least three times.

### Autophagy flux activity detection

Cells (2 × 10^4^ cells per well) were grown on 6-well plates. The mRFP-GFP-LC3 adenovirus vector (GeneChem) was utilized to assess the autophagy activity. Adenovirus infection trial was performed in line with the direction. The autophagic flux was assessed by calculating GFP and mRFP point numbers, and the autophagy flux activity was examined under a confocal microscopy. The experiment was conducted at least three times.

### Luciferase reporter assays

Cells were planted into 96-well plates and transfected with the various luciferase reporter vectors to detect the luciferase activities of Myc pathway, PI3K/AKT pathway, NF-κB pathway, Oct4 pathway, Nanog pathway, Notch pathway, Hippo pathway, Wnt pathway and Hedgehog pathway, respectively. The Cignal Finder Reporter Array (336841, QIAGEN, Germany) was used to identify the signaling pathways regulated by RP1-59D14.5. RP1-59D14.5 cDNA was generated by PCR and inserted into the firefly luciferase plasmid. The luciferase activity was measured by Dual-luciferase reporter assay system (Promega). Firefly luciferase activity was normalized to that of Renilla luciferase.

In addition, RP1-59D14.5 or LATS1/2 fragment covering miR-147a wild-type (Wt) or mutant (Mut)-type binding sites was inserted into pmirGLO luciferase reporter vectors (Promega), and then co-transfected with miR-147a mimics or NC mimics into cells. After 48 h transfection, all luciferase activities were examined by use of luciferase reporter assay system (Promega). The experiment was conducted at least three times.

### Western blot

Total protein was extracted with RIPA Lysis and Extraction Buffer (Thermo Fisher Scientific). The protein concentration was determined using BCA Protein Assay Kit (Abcam, Cambridge, UK). Then the protein extracts were separated by SDS-PAGE and transferred onto a PVDF membrane (Millipore, Billerica, MA, USA). Membranes were blocked with 5% skim milk and incubated with primary antibodies against p62 (Abcam, 1/10000-1/50000), LAMP1 (Abcam, 1/1000-1/10000), LC3 (Abcam, 1/2000), TAZ (Abcam, 1/50-1/200), LATS1 (Abcam, 1/5000), LATS2 (Abcam, 1 µg/ml), CK1 (Abcam, 1 µg/ml), YAP (Abcam, 1/5000), p-YAP (Abcam, 1/10000), GAPDH (CST, 1/1000), HUR (Abcam, 1/1000) and β-actin (Abcam, 1 µg/ml) for 4 °C overnight. Then, the membranes were hatched with horseradish peroxidase-labeled secondary antibodies for 2 hours at room temperature. After being washed again, the protein detections were used ECL Western Blotting Substrate (Invitrogen). The experiment was conducted at least three times. Original western blots are included in Supplemental Material.

### IF (immunofluorescence)

LNCaP and PC3 cells were subjected to treatment with 4% paraformaldehyde for fixation. Subsequently, they were blockaded by 3% BSA in 0.5% Triton X-100. Later, the cells were incubated overnight at 4 °C with primary antibodies against YAP and TAZ. Afterwards, the secondary antibodies conjugated with fluorescence were incubated with cells for 2 hours. DAPI solution was used to counterstain the nuclei of LNCaP and PC3 cells. The fluorescence intensity of cells was detected by fluorescent microscope and quantified by ImageJ. This assay was carried out at least three times.

### RIP

RIP experiment was conducted via the Magna RIP RNA Binding Protein Immunoprecipitation Kit (Millipore, Billerica, MA, USA) based on the manufacturer’s instructions. Cells were lysed and 100 μl of the cell lysates were hatched with RIPA buffer containing magnetic beads conjugated with human anti-Argonaute2 (Ago2) antibody (Millipore) or anti-HUR (Abcam). Normal mouse IgG (Millipore) was served as a negative control. After being washed, cells were hatched with proteinase K. RNA was extracted and analyzed via RT-qPCR. The experiment was conducted at least three times.

### RNA pull-down assays

RNA pull-down assays were performed using a Magnetic RNA Protein Pull-Down Kit (Pierce, USA) on the basis of the manual. Biotinylated RP1-59D14.5 and wild-type or mutant-type biotinylated miR-147a (bio-miR-147a-Wt or bio-miR-147a-Mut) were synthesized by RiboBio (Guangzhou, China). For RNA-protein pull-down assay, biotinylated RP1-59D14.5 were incubated with prewashed streptavidin-agarose beads. Then, RNA-bound beads were incubated with lysates. After being washed and eluted, the protein was subjected to separation on SDS-PAGE and silver staining, followed by western blot.

For RNA-RNA pull-down assay, bio-miR-147a-Wt, or bio-miR-147a-Mut were hatched with cells and cross-linked by 1% paraformaldehyde. Then glycine was used for stopping crosslink. After being washed, the cells and added with DNase I and streptomyces avidin magnetic beads. Finally, protease K was added and the RNA was extracted by Trizol. RT-qPCR was used for analysis. The experiment was conducted at least three times.

### RNA fluorescence in situ hybridization (FISH)

FISH assays were conducted using FISH Kit (RiboBio) in accordance with the protocol. RP1-59D14.5 probes were designed and synthesized by RiboBio. Cells were fixed with 4% paraformaldehyde and permeabilized with 0.5% Triton X-100 at 4 °C for 30 min. Next, cells were subjected to pre-hybridization and added with RP1-59D14.5 probe at 37 °C overnight in the dark. Finally, cells were counterstained with Hoechst and imaged by fluorescence microscope. The experiment was conducted at least three times.

### Xenografts assays

A total of 25 BALB/C nude mice (4 weeks old) were purchased from Vital River Laboratory Animal Technology Co., LTD. (Beijing, China) and fed under specific pathogen-free situations. The nude mice were randomly divided into two groups. Next, cells (1 × 10^6^) were subcutaneously inoculated into the nude mice. One group was injected with pcDNA3.1/RP1-59D14.5 transfected cells and the other group with those transfected with pcDNA3.1. The tumor volume and tumor weight were measured every four days. The nude mice were euthanized on the 28th day, with their tumors being excised. The animal experiment was approved by the Affiliated Huai’an No.1 People’s Hospital of Nanjing Medical University. The experiment was conducted at least three times.

### Bioinformatic analysis

LncBase Predicted v.2 (http://carolina.imis.athena-innovation.gr) was applied for the prediction of miRNAs which might bind to RP1-59D14.5 (Threshold=0.7). Combined with starBase (http://starbase.sysu.edu.cn) database, the mutual miRNA combined with RP1-59D14.5 and LATS1/2 was sifted out. The expression of RP1-59D14.5 in prostate adenocarcinoma (PRAD) tissues was analyzed by GEPIA2 (http://gepia2.cancer-pku.cn) database.

### Statistical analyses

Each experiment was conducted in triplicate. All measurement data were exhibited as mean ± standard deviation (S.D.). Comparisons between two groups were processed with Student’s t-test, and one-way ANOVA was applied for multiple groups, by the application of SPSS 19.0 software (IBM, Armonk, NY, USA). The p-value less than 0.05 were considered as statistical difference.

## Results

### RP1-59D14.5 inhibits cell proliferation, invasion, migration and promotes cell autophagy in PCa

**Fig. 1:**
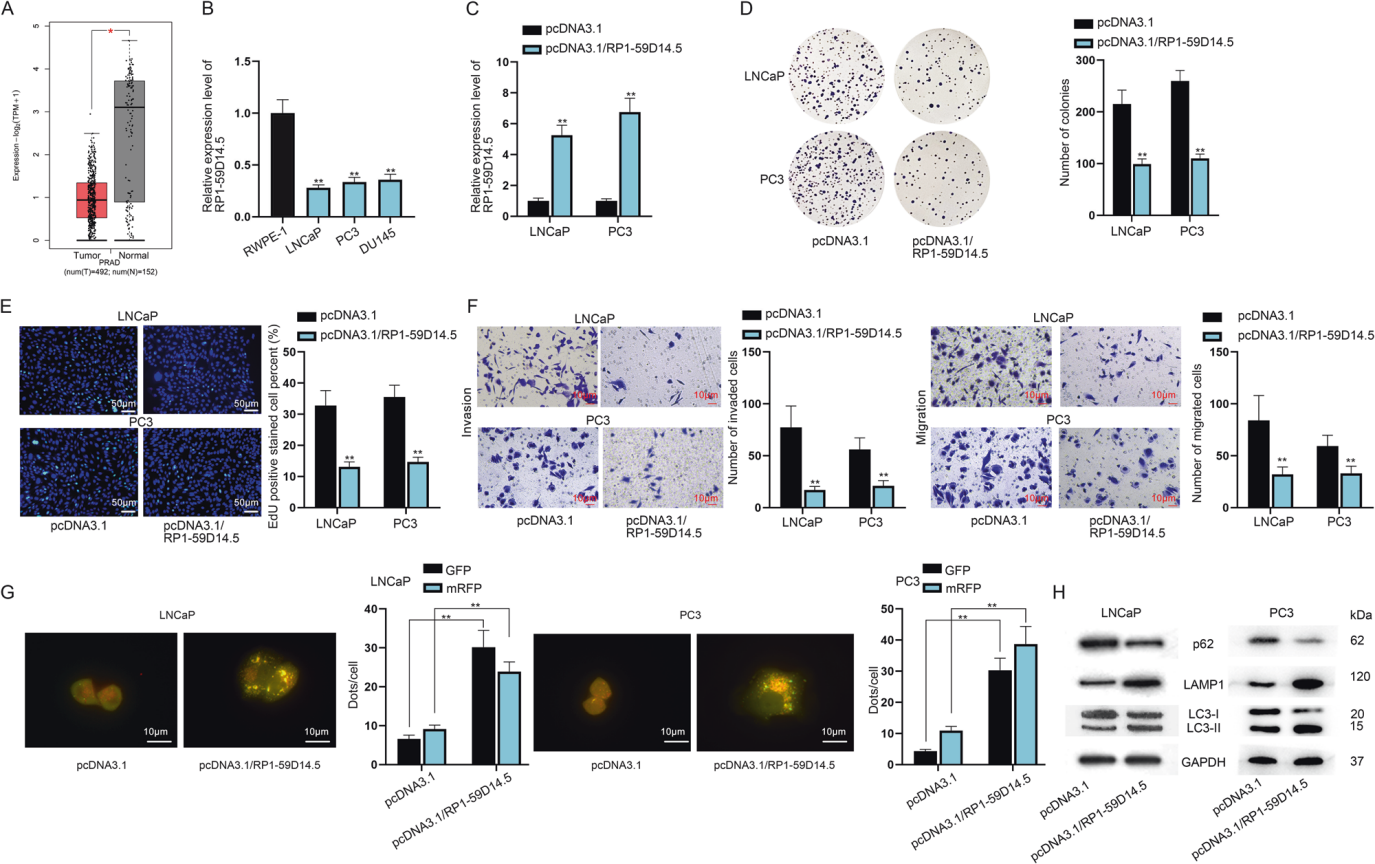
RP1-59D14.5 inhibits cell proliferation, migration and invasion while promotes cell autophagy in PCa. **A** Box plot displayed RP1-59D14.5 expression in PRAD tissues from GEPIA2. T: tumor; N: normal. **B** RT-qPCR analyzed RP1-59D14.5 expression in PCa cell lines (LNCaP, PC3, and DU145) and REPE-1 cell. **C** Overexpression of RP1-59D14.5 was verified via RT-qPCR. **D**, **E** Colony formation and EdU assays assessed the proliferation of RP1-59D14.5-overexpressed PCa cells. Scale bar=50 μm. **F**. Transwell assays detected the invasion and migration of RP1-59D14.5-overexpressed PCa cells. Scale bar = 10 μm. **G** IF analysis detected autophagy flux. The dots of GFP and mRFP in RP1-59D14.5-overexpressed PCa cells were measured by RT-qPCR. Scale bar = 10 μm. **H** Western blot detected autophagy-related markers (LC3-I/LC3-II, p62 and LAMP1) in RP1-59D14.5-overexpressed PCa cells. ^**^P < 0.01.

To investigate the association between RP1-59D14.5 and PCa, we first applied GEPIA2 (http://gepia2.cancer-pku.cn) database to analyze RP1-59D14.5 expression in prostate adenocarcinoma (PRAD) tissues. Compared to normal tissues, RP1-59D14.5 displayed a low expression level in PRAD tissues (Fig. 1A, num (T) = 492; num (N) = 152). Thus, we further detected RP1-59D14.5 expression in PCa cell lines. As shown in Fig. 1B, RP1-59D14.5 expression was significantly down-regulated in PCa cell lines relative to human normal prostatic epithelial cell line (RWPE-1). Then, we selected LNCaP and PC3 cells for follow-up experiments for both cells had a relatively lower expression level of RP1-59D14.5. To further determine the function of RP1-59D14.5 in PCa, RT-qPCR was used to verify the successful overexpression of RP1-59D14.5 in LNCaP and PC3 cells (Fig. 1C). RP1-59D14.5 overexpression obviously reduced the number of colonies in PCa cells (Fig. 1D). Likewise, the proliferation ability was weakened when RP1-59D14.5 was up-regulated (Fig. 1E). Besides, invaded and migrated cells were reduced in RP1-59D14.5 up-regulated PCa cells through transwell assays (Fig. 1F). To further detect autophagy flux, PCa cells were transfected with GFP-mRFP-LC3. RP1-59D14.5 overexpression enhanced both red and yellow puncta in PCa cells, indicating that RP1-59D14.5 promotes autophagy flux (Fig. 1G). After RP1-59D14.5 upregulation, protein levels of LC3-II and LAMP1 were remarkably increased by RP1-59D14.5 overexpression, whereas that of p62 and LC3-I protein levels were markedly downregulated (Fig. 1H). On the other hand, we investigated the loss-of-function effects of RP1-59D14.5 in normal RWPE-1 cells. The knockdown efficiency of sh/RP1-59D14.5#1/2/3 was elucidated via RT-qPCR (Supplementary Fig. S1A). It was found that colonies, EdU positive stained cells, invaded and migrated cells were overtly increased with RP1-59D14.5 silencing (Supplementary Fig. S1B-D). In addition, the dots of GFP and mRFP were sharply reduced with RP1-59D14.5 knockdown (Supplementary Fig. S1E). Western blot analysis showed that p62 and LC3-I protein levels were increased while LAMP1 and LC3-II protein levels were diminished after the ablation of RP1-59D14.5 (Supplementary Fig. S1F). Totally, RP1-59D14.5 inhibited cell proliferation, invasion, migration and promoted cell autophagy in PCa.

### RP1-59D14.5 activates the Hippo pathway in PCa cells

**Fig. 2:**
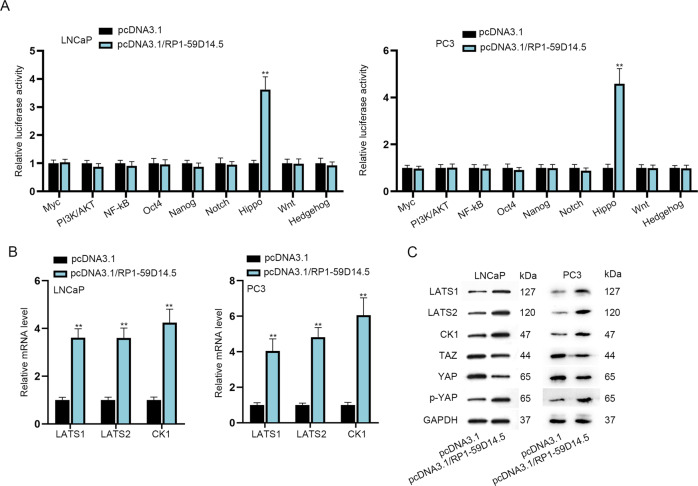
RP1-59D14.5 activates the Hippo pathway in PCa cells. **A** The activities of various pathways were detected by luciferase reporter assays. **B**, **C** RT-qPCR was used to detect mRNA levels of LATS1/2, CK1, and western blot analyzing protein levels of LATS1/2, CK1, TAZ, YAP, and p-YAP in RP1-59D14.5-overexpressed PCa cells. ^**^P < 0.01.

As we know, lncRNAs are implicated in the development of human cancers via regulating various signaling pathways [19]. Herein, we supposed that whether RP1-59D14.5 exerts its anticancer functions via modulating certain signaling pathway. As revealed in Fig. 2A, RP1-59D14.5 up-regulation obviously elevated the luciferase activity of the Hippo pathway but barely affected that of other pathways. Then, RP1-59D14.5 up-regulation led to an increase in mRNA and protein levels of LATS1/2, CK1 and the protein level of phosphorylated YAP (p-YAP), and to a decrease in the protein level of TAZ and YAP (Fig. 2B, C). Furthermore, IF showed that relative fluorescence intensity of TAZ or YAP in cytoplasm and nucleus was largely reduced with RP1-59D14.5 silencing (Supplementary Fig. S2A). RP1-59D14.5 overexpression inhibited the transcription activity of YAP/TAZ (Supplementary Fig. S2B). All these results suggested that RP1-59D14.5 activated the Hippo pathway in PCa cells.

### RP1-59D14.5 targets the miR-147a/LATS1/2 axis in PCa cells

**Fig. 3:**
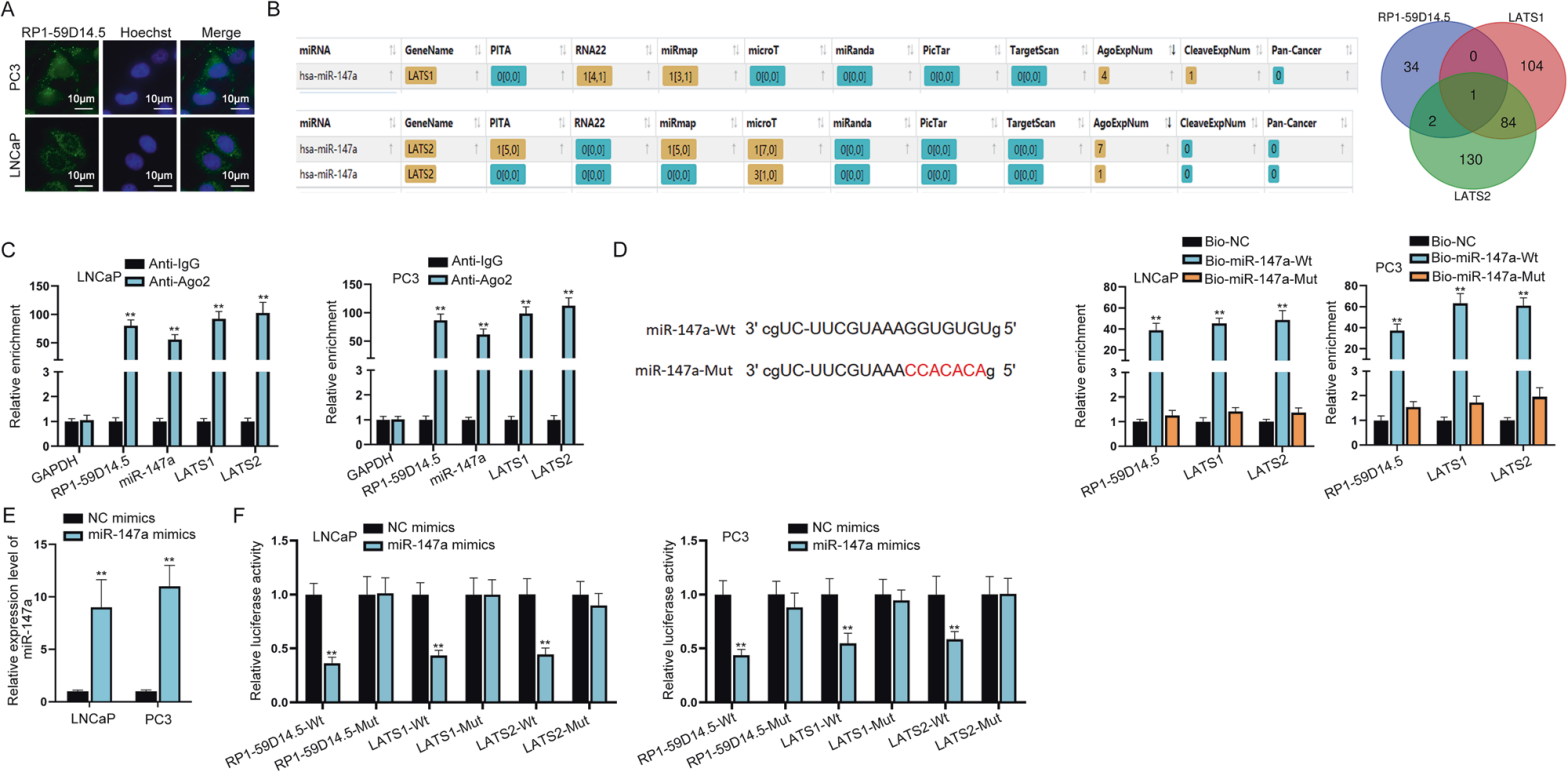
RP1-59D14.5 targets the miR-147a/LATS1/2 axis in PCa cells. **A** FISH assays determined the subcellular location of RP1-59D14.5 in PCa cells. Scale bar = 10 μm. **B** The starBase prediction of putative miRNAs of LATS1/2. Venn diagram illustrated a common miRNA of RP1-59D14.5 and LATS1/2. **C** RIP assays were performed to detect the association of RP1-59D14.5, miR-147a, and LATS1/2 with Ago2-RISC. **D** The sequence of miR-147a-Wt (wild type miR-147a) and miR-147a-Mut (mutant miR-147a) were shown. RNA pull down assays detected the enrichment of RP1-59D14.5 and LATS1/2. **E** RT-qPCR examined miR-147a expression in PCa cells transfected with miR-147a mimics. **F** Luciferase reporter assays detected the luciferase activity of wild-type or mutant-type RP1-59D14.5 and LATS1/2. ^**^P < 0.01.

Next, we explored the mechanism of RP1-59D14.5 through regulating the Hippo pathway in PCa cells. First, FISH assays indicated that RP1-59D14.5 was mainly distributed in the cytoplasm of PCa cells, which suggested that RP1-59D14.5 has the potential to act as a ceRNA to regulate gene expression at a post-transcriptional level (Fig. 3A). Accordingly, we applied starBase (http://starbase.sysu.edu.cn) and LncBase Predicted v.2 (http://carolina.imis.athena-innovation.gr) databases to screen the mutual miRNA of RP1-59D14.5 and LATS1/2 (Fig. 3B, left panel). Venn diagram illustrated that miR-147a was the target miRNA (Fig. 3B, right panel). Next, we found that RP1-59D14.5, miR-147a and LATS1/2 were highly enriched in the pull-down complexes by Anti-Ago2, indicating the involvement of RP1-59D14.5, miR-147a, and LATS1/2 in ceRNA network (Fig. 3C and Supplementary Fig. S3A). As shown in Fig. 3D, miR-147a-Mut was acquired by mutating the 5’ terminal nucleotide (nt) 2–8 of miR-147a. RNA pull down assays further proved that RP1-59D14.5 and LATS1/2 were largely abundant in bio-miR-147a-Wt groups while bio-miR-147a-Mut had no marked change (Fig. 3D). In addition, we stably up-regulated miR-147a expression in PCa cells (Fig. 3E). The data indicated that miR-147a overexpression evidently reduced the luciferase activity of RP1-59D14.5-Wt and LATS1/2-Wt, while that of RP1-59D14.5-Mut and LATS1/2-Mut had no significant difference (Fig. 3F). The decreased LATS1/2 expression caused by miR-147a overexpression could be increased by RP1-59D14.5 overexpression (Supplementary Fig. S3B, C). Meanwhile, RP1-59D14.5 knockdown led to a marked reduction on LATS1/2 expression (Supplementary Fig. S3D, E). All these data indicated that RP1-59D14.5 acted as a ceRNA to target the miR-147a/LATS1/2 axis in PCa cells.

### MiR-147a promoted PCa cell growth, invasion and migration but inhibited autophagy

Moreover, we explored the effect of miR-147a on PCa progression. Through proliferation assays, it was found that miR-147a overexpression caused an increase on the number of colonies and EdU positive stained cells (Supplementary Fig. S4A, B). Similarly, invaded and migrated cells were markedly increased by upregulated miR-147a (Supplementary Fig. S4C). According to IF and western blot analyses, miR-147a mimics repressed autophagy in PCa since the dots of GFP and mRFP were largely reduced (Supplementary Fig. S4D), and LAMP1 and LC3-II protein levels were diminished and p62 and LC3-I protein levels were increased (Supplementary Fig. S4E). Altogether, miR-147a enhanced cell growth, invasion, and migration but suppressed autophagy in PCa.

### RP1-59D14.5 affects PCa cell progression via targeting LATS1/LATS2/miR-147a

**Fig. 4:**
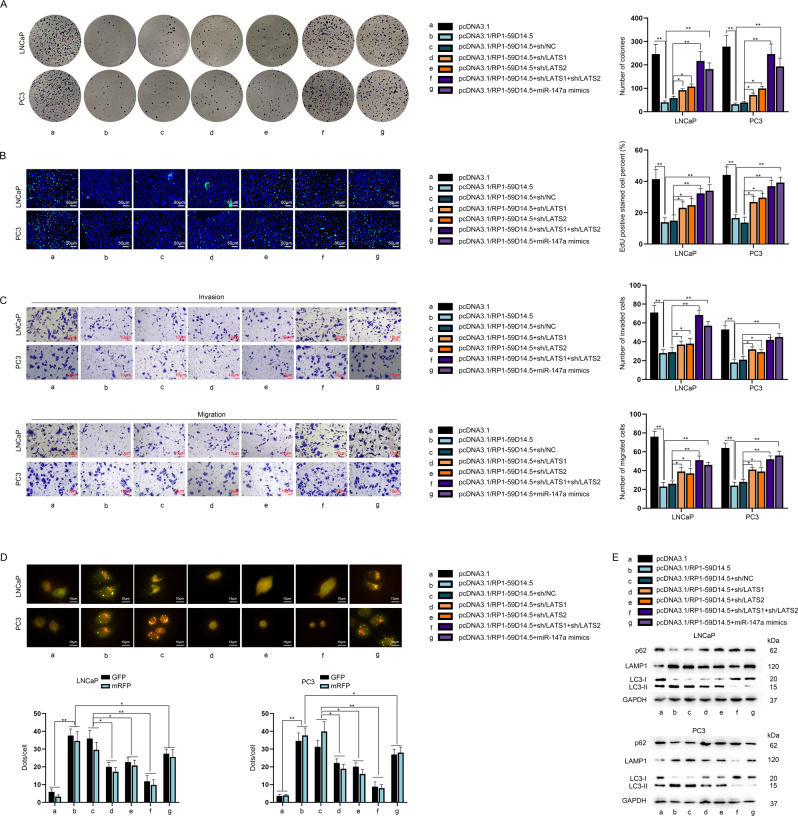
RP1-59D14.5 affects PCa cell progression via targeting LATS1/LATS2/miR-147a. **A**, **B** Colony formation and EdU assays assessed the proliferation of the indicated transfected cells. Scale bar = 50 μm **C** Transwell assays detected the invasion and migration of the indicated transfected cells. Scale bar = 10 μm. **D** The dots of GFP and mRFP in differently transfected cells were detected by IF analysis and quantified by RT-qPCR. Scale bar = 10 μm. **E** Western blot measured the protein levels of p62, LAMP1, LC3-I/II. ^*^P < 0.05, ^**^P < 0.01.

To validate the interaction of RP1-59D14.5 and miR-147a in PCa cells, rescue experiments were carried on. The number of colonies and EdU positive stained cells was reduced with RP1-59D14.5 overexpression, while co-transfection of sh/LATS1/2, sh/LATS1 + sh/LATS2 or miR-147a mimics, partially reversed this effect (Fig. 4A, B). Besides, LATS1/2 silencing, LATS1 + LATS2 knockdown or miR-147a overexpression partially restored the inhibition of RP1-59D14.5 overexpression on PCa cell invasion and migration (Fig. 4C). In addition, the enhanced autophagy induced by RP1-59D14.5 overexpression was partially repressed by co-transfection of sh/LATS1/2, sh/LATS1 + sh/LATS2 or miR-147a mimics (Fig. 4D). Western blot analysis displayed that the reduced p62 and LC3-I protein levels and increased levels of LAMP1 and LC3-II caused by upregulated RP1-59D14.5 were partially reversed by depleted LATS1/2, silenced LATS1 + LATS2 or overexpressed miR-147a (Fig. 4E). Collectively, RP1-59D14.5 affected PCa cells progression via targeting LATS1/LATS2/miR-147a.

### RP1-59D14.5 recruits HUR to promote CK1 expression and thereby activates the Hippo pathway in PCa cells

**Fig. 5:**
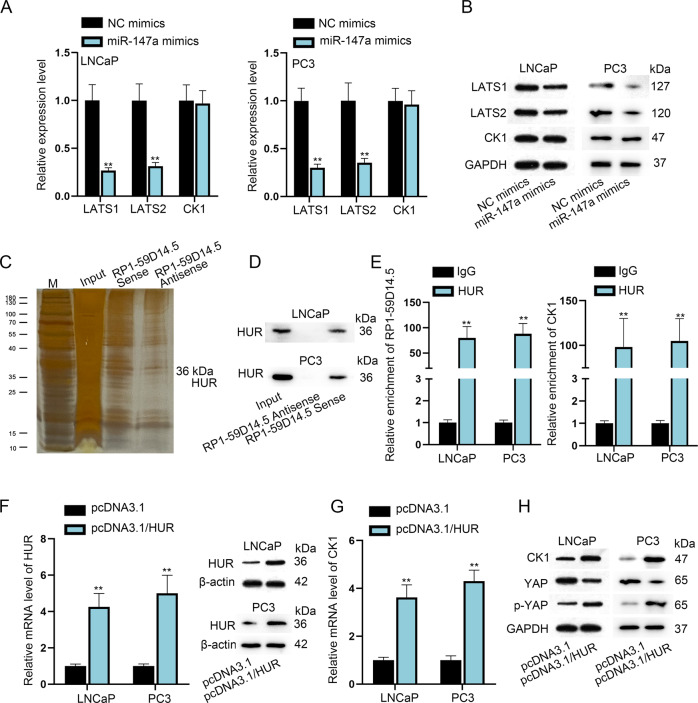
RP1-59D14.5 recruits HUR to promote CK1 expression and activates the Hippo pathway in PCa cells. **A**, **B** RT-qPCR and western blot detected the mRNA and protein levels of LATS1/2 and CK1. **C** RNA pull down assays analyzed the proteins combined with RP1-59D14.5. M: marker. HuR: 36 kDa. **D** RNA pulldown assay analyzed the interaction between HUR and RP1-59D14.5. **E** RIP assays detected the enrichment of RP1-59D14.5 and CK1. **F** RT-qPCR and western blot detected the levels of HUR with HUR overexpression. β-actin serves as an internal control. **G**, **H** RT-qPCR and western blot detected mRNA level of CK1 and protein levels of CK1, YAP and p-YAP in HUR-overexpressed cells. ^**^P < 0.01.

Then, we speculated that the RP1-59D14.5/miR-147a/LATS1/2 axis could not fully activate the Hippo pathway. Thus, we used RT-qPCR and western blot to analyze levels of LATS1/2 and CK1. The results indicated that miR-147a overexpression declined levels of LATS1/2 but had no effects on CK1 expression (Fig. 5A, B). A specific band was indicated in the electrophoretic gel at about 36 kDa compared to RP1-59D14.5 antisense (Fig. 5C). The protein level of HUR was highly enriched in RP1-59D14.5 Sense groups (Fig. 5D). Besides, RIP assays showed that both RP1-59D14.5 and CK1 were enriched in HUR groups (Fig. 5E). Moreover, the overexpression efficiency of pcDNA3.1/HUR was verified (Fig. 5F). We found that mRNA and protein level of CK1 and protein level of phosphorylated YAP (p-YAP) were overtly increased when HUR was up-regulated (Fig. 5G, H). Meanwhile, the level of YAP was reduced with HUR overexpression (Fig. 5H). Taken together, RP1-59D14.5 recruited HUR to promote CK1 expression and thereby activated the Hippo pathway.

### RP1-59D14.5 affects PCa cells progression via regulating CK1

**Fig. 6:**
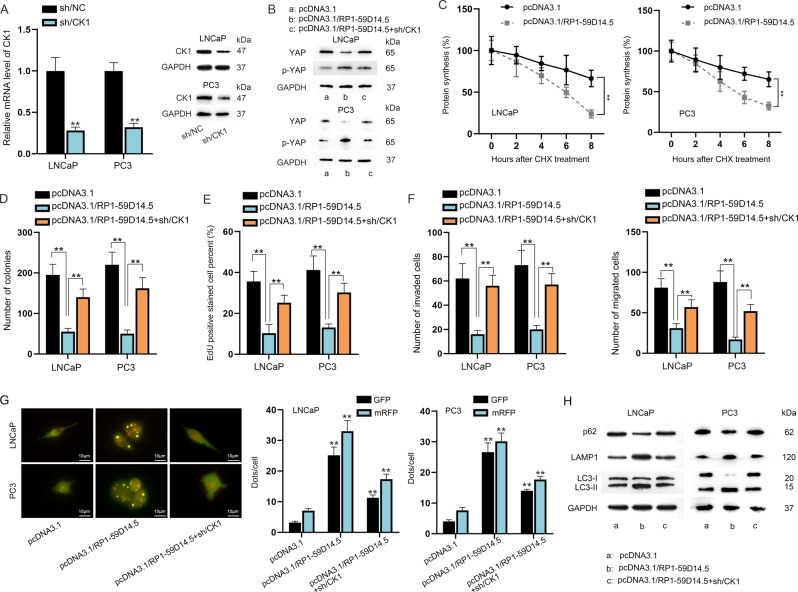
RP1-59D14.5 affects PCa cell progression via regulating CK1. **A** RT-qPCR and western blot detected the level of CK1 in cells transfected with sh/CK1. **B** Rescue experiments were conducted in cells transfected with pcDNA3.1 or pcDNA3.1/RP1-59D14.5 or pcDNA3.1/RP1-59D14.5+sh/CK1. **C** The effect of RP1-59D14.5 overexpression on protein synthesis of YAP was evaluated after CHX treatment. **D**, **E** Colony formation and EdU assays assessed the proliferation of PCa cells. **F** Transwell assays detected the invasion and migration of transfected PCa cells. **G** The dots of GFP and mRFP in differently transfected cells were detected via IF and quantified via RT-qPCR. Scale bar = 10 μm. **H** Western blot measured the levels of p62, LAMP1, LC3-I/II. GAPDH serves as an internal control. ^**^P < 0.01.

It has been reported that CK1 can phosphorylate YAP and then recruit βTrCP to catalyze YAP ubiquitination, ultimately leading to YAP degradation [20]. To investigate the interaction between CK1 and RP1-59D14.5, we stably silenced CK1 (Fig. 6A). Reduced YAP expression induced by RP1-59D14.5 overexpression was reversed by CK1 knockdown while increased p-YAP expression caused by upregulated RP1-59D14.5 was reversed by silenced CK1 (Fig. 6B). As shown in Fig. 6C, protein synthesis of YAP was inhibited by increased RP1-59D14.5. The inhibited proliferation ability mediated by RP1-59D14.5 overexpression was partially restored by co-transfection of sh/CK1 (Fig. 6D, E and Supplementary Fig. S5A, B). In transwell assays, CK1 interference reversed the repressed invasive and migratory capacities caused by elevated RP1-59D14.5 (Fig. 6Fand Supplementary Fig. S5C). In addition, RP1-59D14.5 overexpression enhanced the autophagy in PCa cells, while this effect was partially abrogated by CK1 knockdown (Fig. 6G). The results of western blot concerning autophagy-related proteins further proved it (Fig. 6H). In short, RP1-59D14.5 affected PCa cell proliferation, migration, invasion and autophagy via regulating CK1.

### RP1-59D14.5 inhibits tumor growth in PCa

**Fig. 7:**
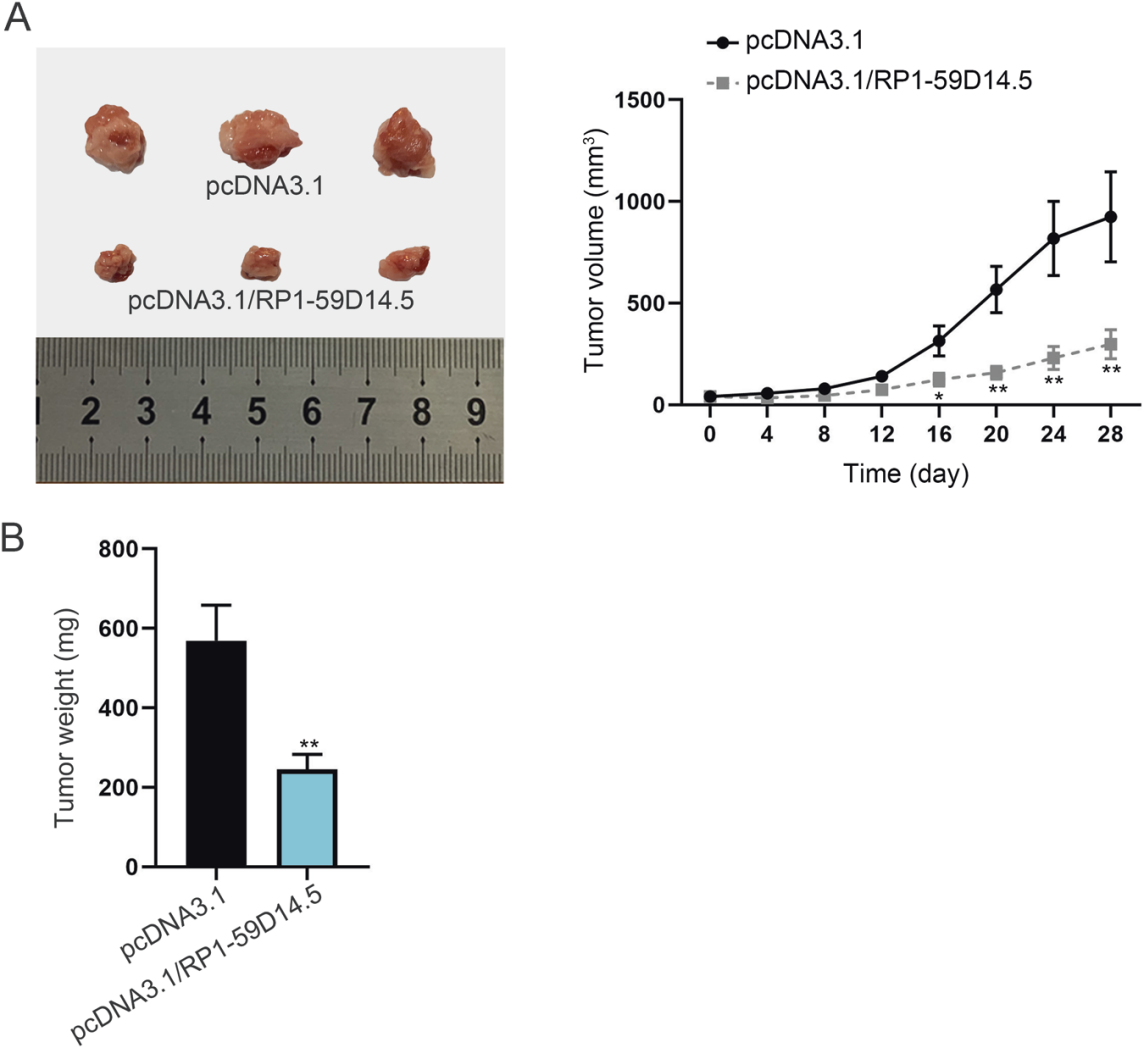
RP1-59D14.5 inhibits tumor growth in PCa. **A**, **B** Images of xenografted tumors excised from the mice injected with pcDNA3.1- or pcDNA3.1/RP1-59D14.5-transfected PCa cells. Tumor volume and weight were measured. ^**^P < 0.01.

Finally, we performed in vivo experiments to validate the function of RP1-59D14.5. As shown in Fig. 7A, B, the tumor volume and tumor weight were significantly reduced when RP1-59D14.5 was upregulated. A conclusion drew that RP1-59D14.5 inhibited PCa tumor growth in vivo.

### A working model displays the mechanism of which RP1-59D14.5-modulated PCa tumorigenesis and progression

**Fig. 8:**
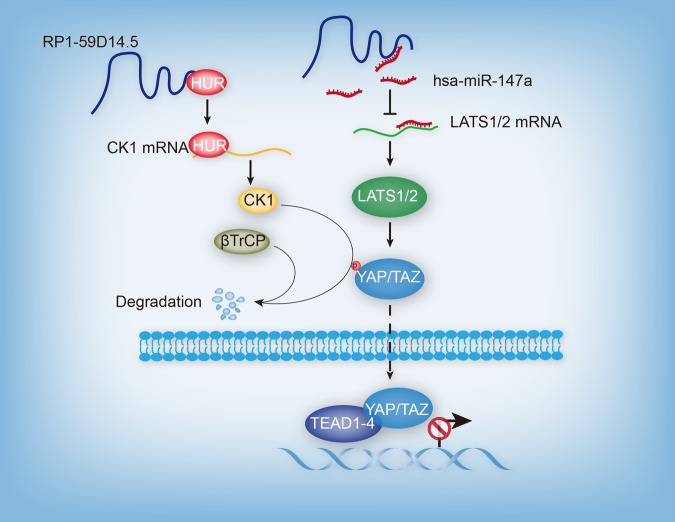
A working model displays the mechanism of which RP1-59D14.5-modulated PCa tumorigenesis and progression. RP1-59D14.5 targets miR-147a/LATS1/2 axis as a ceRNA or recruits HUR to upregulate CK1 which could phosphorylate YAP and then recruit βTrCP to promote YAP degradation, consequently activating the Hippo signaling pathway in PCa cell.

RP1-59D14.5 promoted YAP degradation to activate the Hippo pathway in PCa progression via targeting the miR-147a/LATS1/2 axis and recruiting HUR to promote the interaction of CK1 and βTrCP (Fig. 8).

## Discussion

Recently, accumulating evidences have indicated the important role of lncRNAs in the tumorigenesis and progression of PCa [21]. In our study, we identified that RP1-59D14.5 exhibits a poor expression level in PCa cells. Besides, overexpression of RP1-59D14.5 repressed cell proliferation, migration, and invasion while enhanced cell autophagy in PCa. Consistently, lncRNA PCAT29 serves as an androgen-modulated tumor suppressor in PCa and represses cell proliferation and migration [22]. LncRNA H19 targets miR-675 to inhibit PCa metastases [23]. All these findings supported the tumor-inhibiting role of RP1-59D14.5 in PCa, and RP1-59D14.5 might be a potential target for PCa treatment.

Our study further demonstrated that RP1-59D14.5 activated the Hippo pathway in PCa cells. It has been reported that CD44 elevates the capacities of migration and invasion in docetaxel-resistant PCa cells via induction of the Hippo-YAP signaling pathway [24]. YAP is the pivotal component of the Hippo pathway and has been well documented for its involvement in organ size control and stem cell differentiation [25, 26]. LATS-mediated phosphorylation of YAP leads to the down-regulated expression of YAP via increasing its cytoplasmic accumulation and ubiquitination along with subsequent proteasomal degradation [20]. When the Hippo pathway turns on, it can inhibit cell growth and proliferation by phosphorylating and inhibiting YAP. Inversely, when the Hippo pathway is deactivated, YAP is dephosphorylated and transfers into the nucleus, where it bind to TEAD family and facilitates proliferation and migration of cancer cells [16]. In our study, it was confirmed that RP1-59D14.5 activated the Hippo pathway via regulating LATS1/2 expression as well as YAP phosphorylation and total YAP protein levels. Furthermore, we found that RP1-59D14.5 was mainly distributed in the cytoplasm of the PCa cells, indicating that it exerted functions in post-transcriptional regulation. Meanwhile, increasing evidences have uncovered that lncRNAs can act as ceRNAs to target miRNAs in cancer [27]. Therefore, this research further confirmed that RP1-59D14.5 modulated LATS1/2 expression via targeting miR-147a and then activated the Hippo pathway. In a word, the existence of a regulatory network in PCa was found as that RP1-59D14.5 interacted with miR-147a to co-regulate the expression and function of LATS1/2. As reported previously, miR-147a represses the growth and metastasis of non-small-cell lung cancer via targeting CCL5 [28]. In the current study, rescue experiments validated that both miR-147a and LATS1/2 could reverse the inhibited cell proliferation, migration, invasion and the enhanced autophagy caused by RP1-59D14.5 knockdown. In spite of these findings, we also needed to perform more experiments in vivo or in vitro to explore the interactions between RP1-59D14.5 and Hippo pathway in depth.

Further, this study found that RP1-59D14.5 recruited HUR to up-regulate CK1 expression. As is known, CK1 family of serine (Ser)/threonine (Thr) protein kinases takes part in various cellular processes including developmental signaling. CK1 can mediate the phosphorylation of components in the Hedgehog and Wnt pathway [29]. Then, our study found that CK1 could promote the protein level of YAP phosphorylation to activate the Hippo pathway. Besides, functional experiments indicated that RP1-59D14.5 affects PCa cell progression via regulating CK1 expression. It has been reported CK1 can phosphorylate YAP and then recruit βTrCP to catalyze YAP ubiquitination, ultimately leading to YAP degradation [20]. Herein, we confirmed that RP1-59D14.5 recruited HUR to promote the interaction of CK1 and βTrCP and then lead to YAP degradation. More importantly, our study performed in vivo experiments to confirm the antitumor role of RP1-59D14.5 in PC growth.

In conclusion, for the first time, our study verified the down-regulated expression of RP1-59D14.5 in PCa. RP1-59D14.5 was certified to trigger autophagy and repress tumorigenesis and progression of prostate cancer via activation of the Hippo signaling pathway. Our findings may offer a novel insight into the diagnosis and treatment for PCa.

## Supplementary information

Supplementary figure and table legends

Figure S1

Figure S2

Figure S3

Figure S4

Figure S5

Supplementary Table 1

Supplemental Material

Supplemental file

aj-checklist

cddis-author-contribution-form

## Data Availability

The data that support the findings of this study are available from the corresponding author upon reasonable request.
