# Supplementary material for: RP1-59D14.5 triggers autophagy and represses tumorigenesis and progression of prostate cancer via activation of the Hippo signaling pathway
Source: Cell Death Dis. 2022 May 13;13(5):458. doi: 10.1038/s41419-022-04865-y (PMC9106715; doi:10.1038/s41419-022-04865-y)
Supplement: Supplementary file 1 — Supplementary figure and table legends [file 41419_2022_4865_MOESM1_ESM.pdf]

Figure S1. A. The knockdown of sh/RP1-59D14.5#1/2/3 was validated via qRT-PCR. **B-C.** The loss-of-function effect of RP1-59D14.5 on cell proliferation was evaluated by colony formation and EdU assays. Scale bar=50 μm. **D.** The loss-of-function effects of RP1-59D14.5 on the invasion and migration of RWPE-1 cells were assessed by transwell assays. Scale bar=10 μm. **E.** Autophagy flux was detected after sh/RP1-59D14.5#1/2 treatment. Scale bar=10 μm. **F.** Western blot was applied to measure levels of autophagy-related markers (LC3-I/LC3-II, p62 and LAMP1). GAPDH serves as an internal control. $^{**}P<0.01$.

Figure S2. A. IF analysis was applied to detect the localization of YAP/TAZ after RP1-59D14.5 overexpression (left panels). Scale bar=10 μm. Quantification of IF analysis results was shown (right panels). **B.** Luciferase reporter assay was performed to detect the transcription activity of YAP/TAZ with RP1-59D14.5 overexpression. $^{**}P<0.01$.

Figure S3. A. Images of agarose gel electrophoresis (corresponding to Figure 3C) were shown. **B-C.** RT-qPCR and western blot analyses of levels of LATS1/LATS2 respectively. **D-E.** RT-qPCR and western blot analyses of levels of LATS1/LATS2 with RP1-59D14.5 downregulation. $^{**}P<0.01$.

Figure S4. A-B. The effect of miR-147a overexpression on cell growth was analyzed by proliferation assays. Scale bar=50 μm. **C.** The gain-of-function effect of miR-147a on cell invasion and migration was assessed by transwell assays. Scale bar=10 μm. **D.** Autophagy flux was assessed with miR-147a mimics. Scale bar=10 μm. **E.** Western blot was applied to measure levels of autophagy-related markers with miR-147a mimics. GAPDH serves as an internal control. $^{**}P<0.01$.

Figure S5. A-C. Representative images of colony formation, EdU and transwell invasion and migration assays (corresponding to Figure 6D-F) were shown. Scale bar=50 μm (Figure S5B). Scale bar=10 μm (Figure S5C).

Supplementary Table 1. Mass spectrometry analysis of RP1-59D14.5 binding proteins

Supplemental Material. Original data of western blot analysis.
