# Supplementary figures and images for: RP1-59D14.5 triggers autophagy and represses tumorigenesis and progression of prostate cancer via activation of the Hippo signaling pathway
Source: Cell Death Dis. 2022 May 13;13(5):458. doi: 10.1038/s41419-022-04865-y (PMC9106715; doi:10.1038/s41419-022-04865-y)

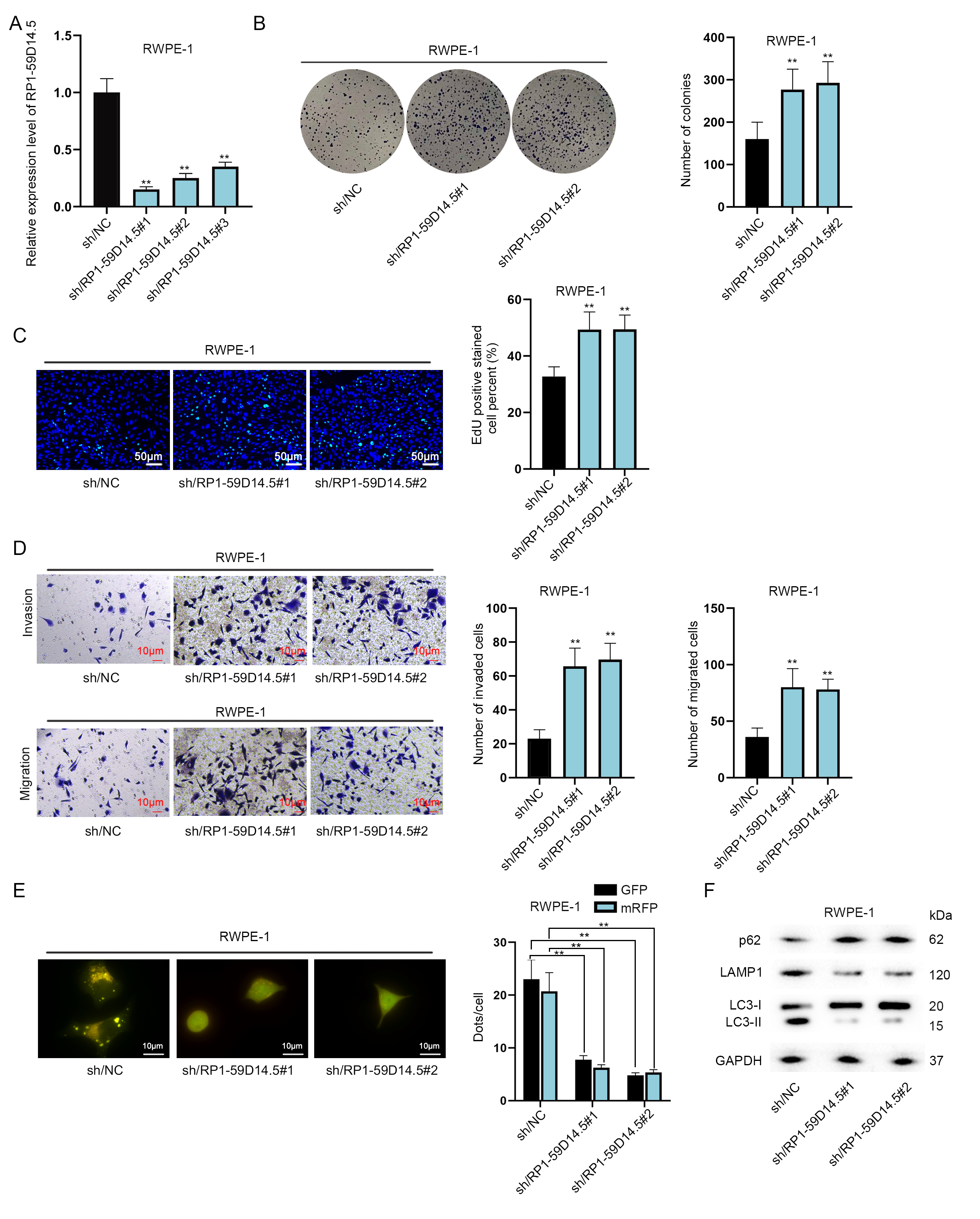

Supplement: Supplementary file 2 — Figure S1 [file 41419_2022_4865_MOESM2_ESM.tif]

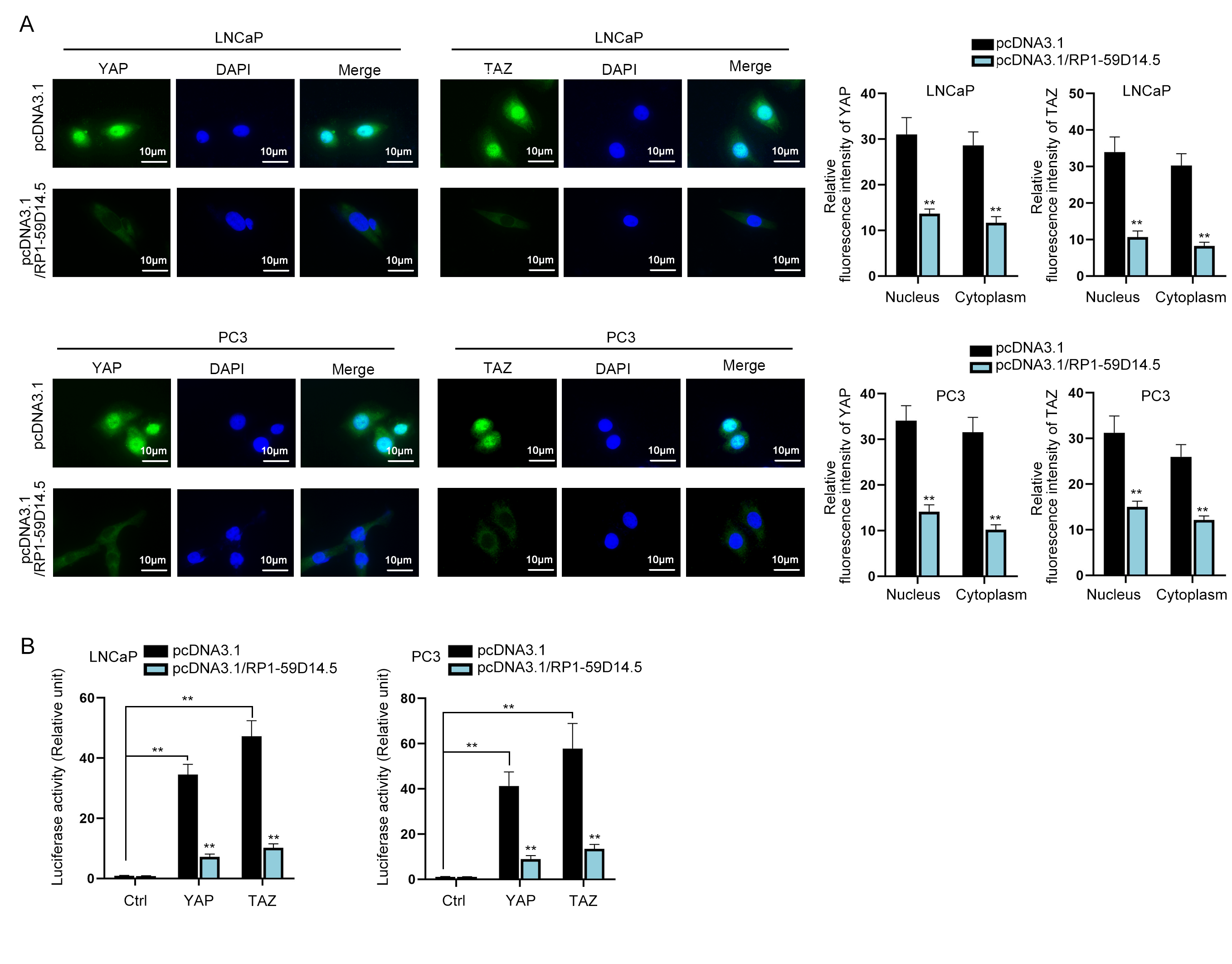

Supplement: Supplementary file 3 — Figure S2 [file 41419_2022_4865_MOESM3_ESM.tif]

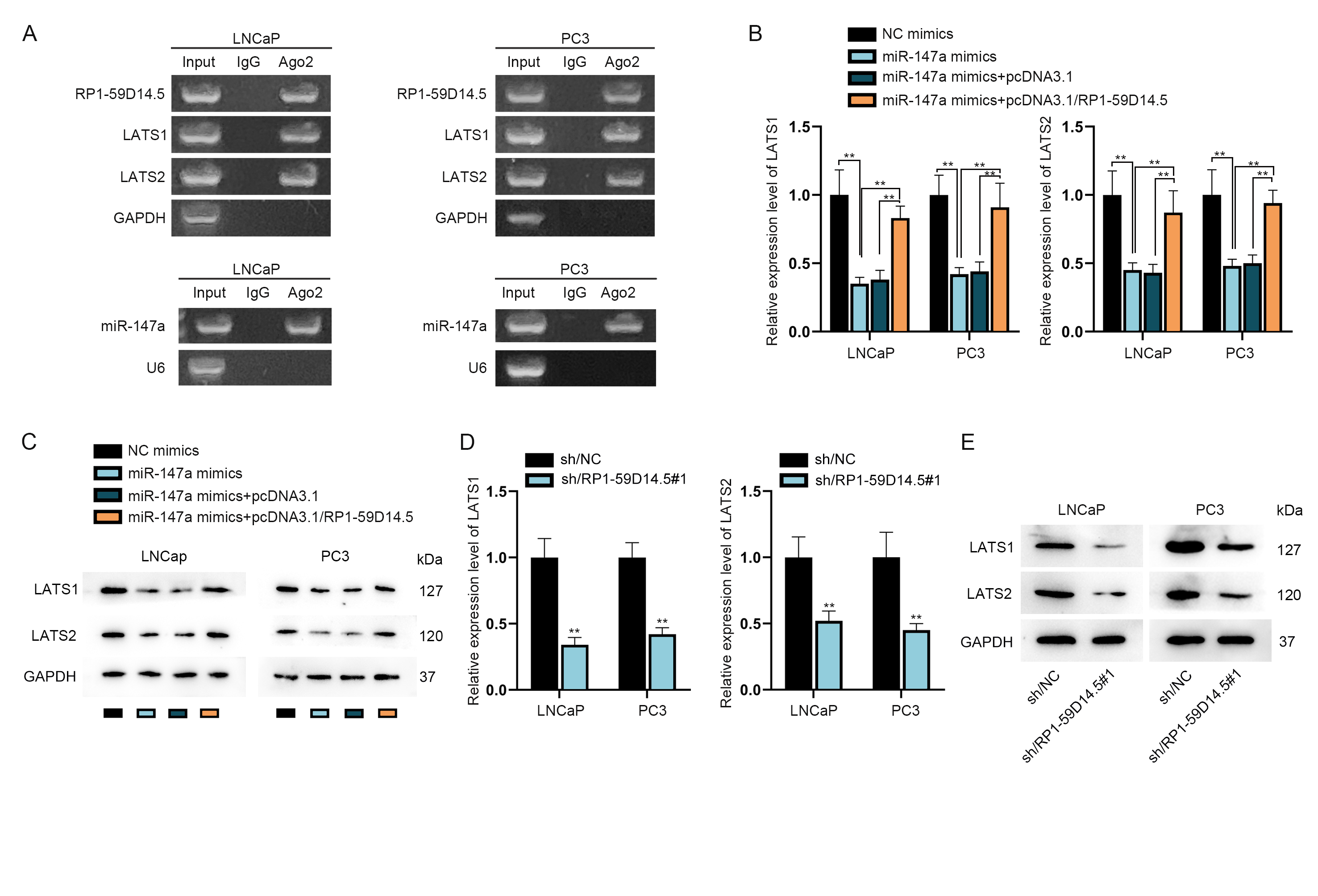

Supplement: Supplementary file 4 — Figure S3 [file 41419_2022_4865_MOESM4_ESM.tif]

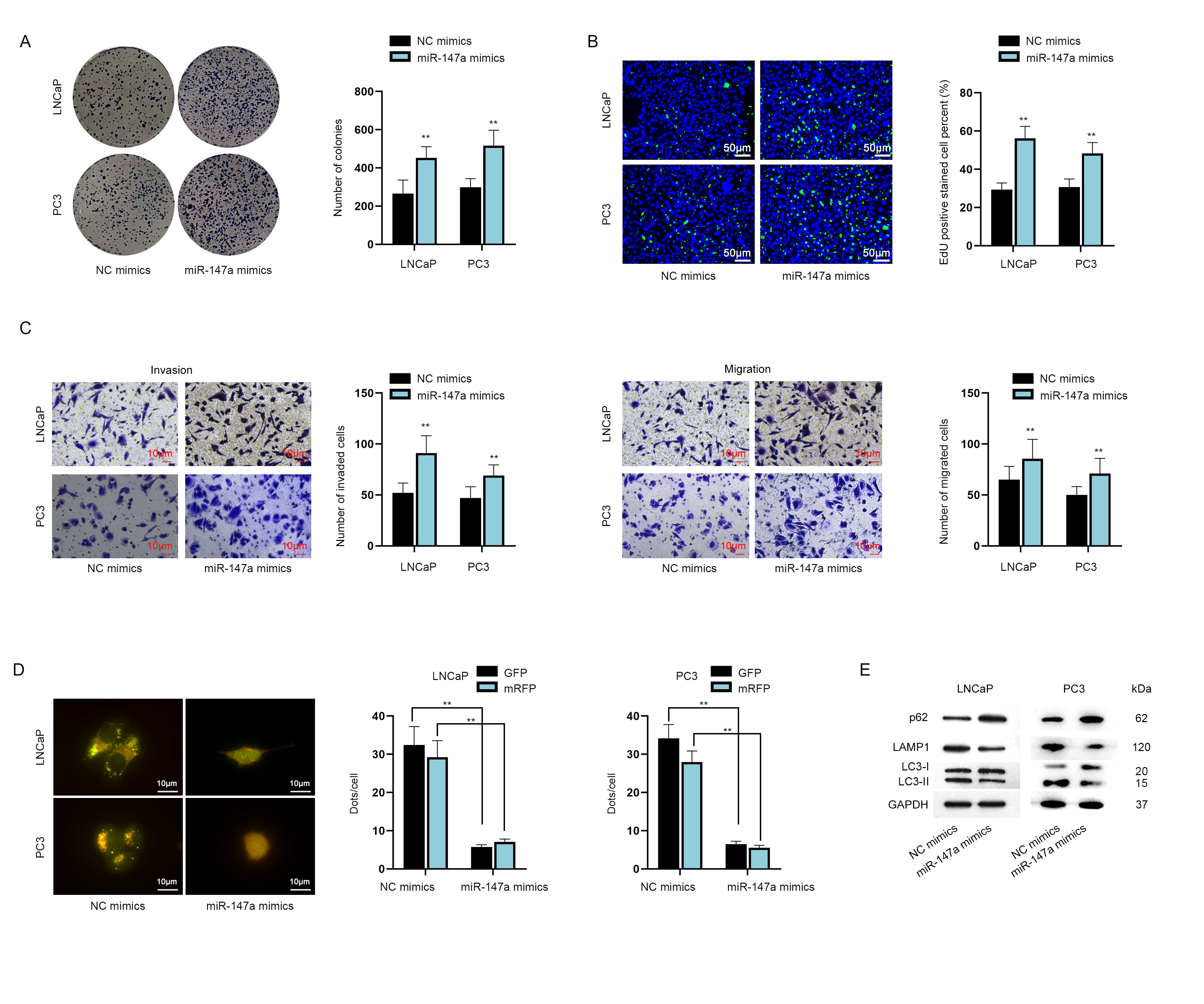

Supplement: Supplementary file 5 — Figure S4 [file 41419_2022_4865_MOESM5_ESM.tif]

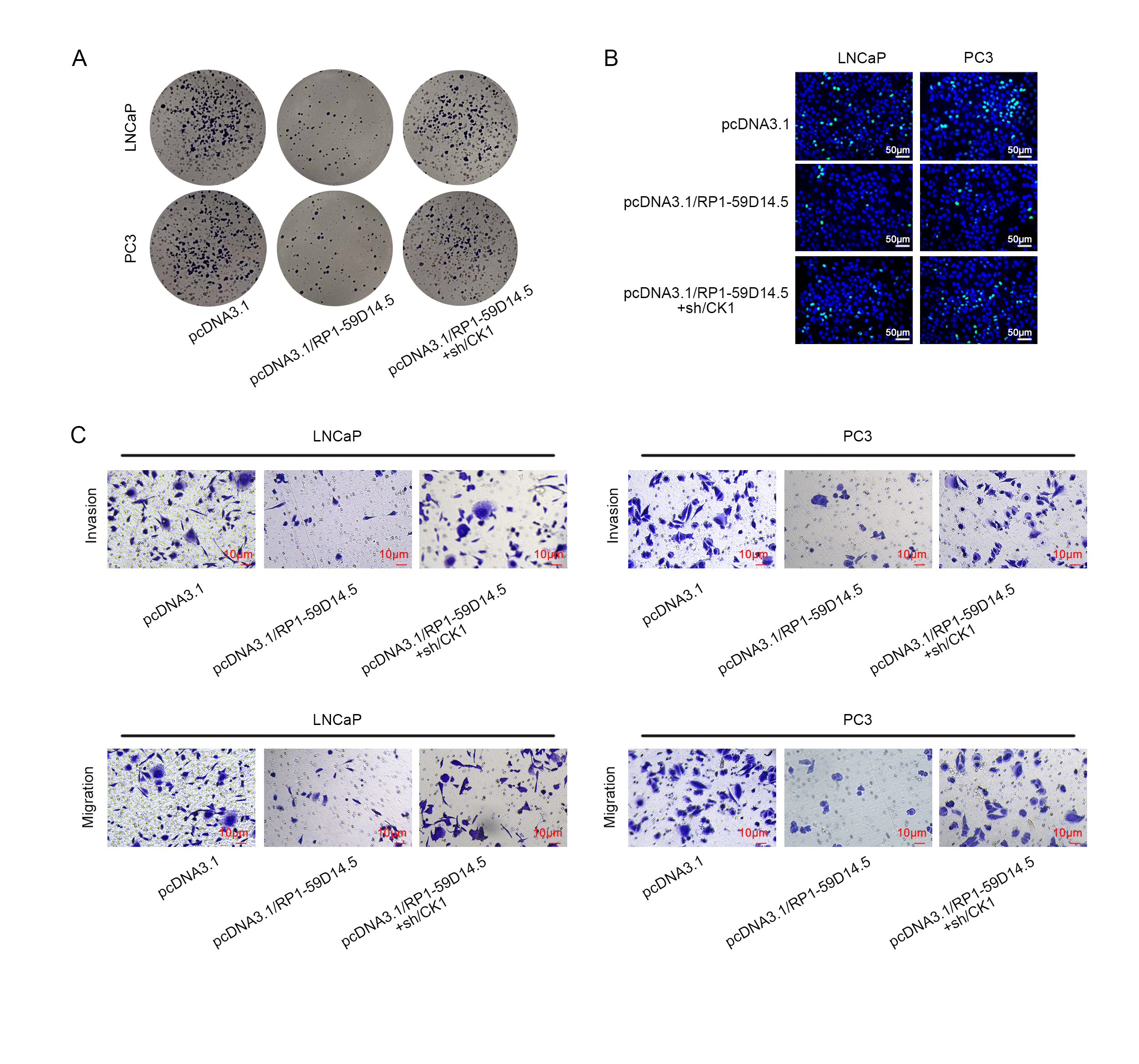

Supplement: Supplementary file 6 — Figure S5 [file 41419_2022_4865_MOESM6_ESM.tif]

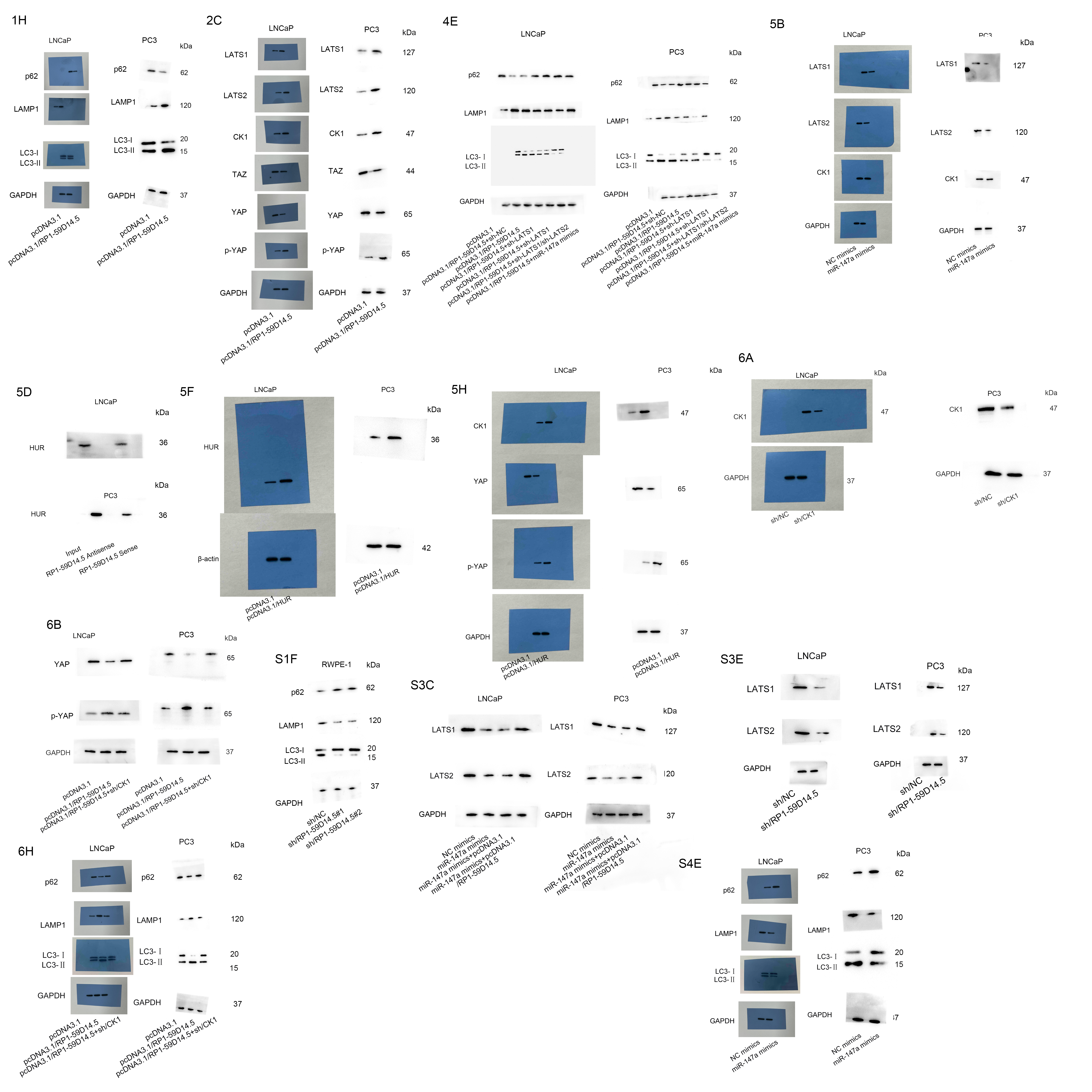

Supplement: Supplementary file 8 — Supplemental Material [file 41419_2022_4865_MOESM8_ESM.tif]

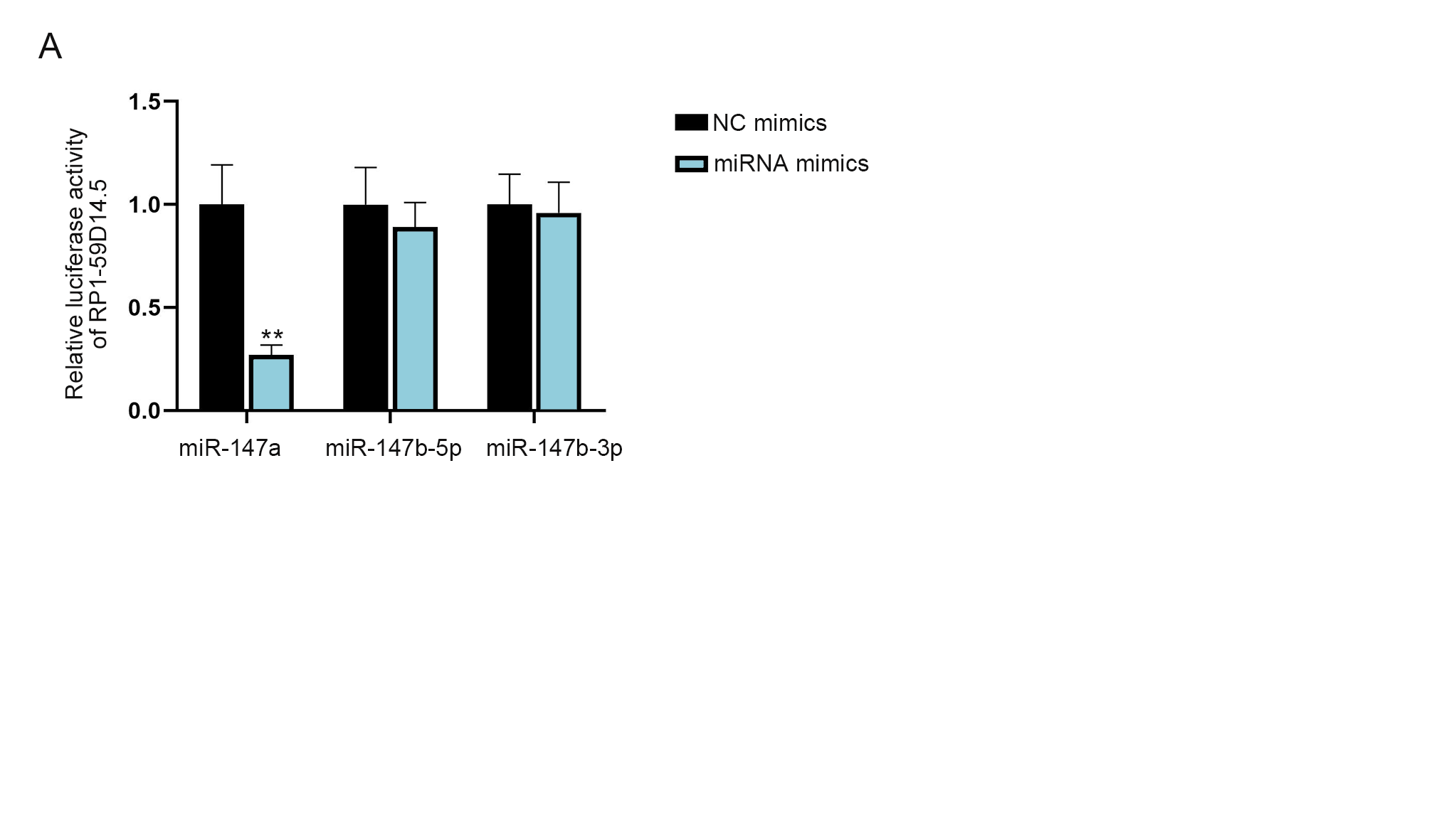

Supplement: Supplementary file 9 — Supplemental file [file 41419_2022_4865_MOESM9_ESM.tif]
