# Supplementary material for: RP1-59D14.5 triggers autophagy and represses tumorigenesis and progression of prostate cancer via activation of the Hippo signaling pathway
Source: Cell Death Dis. 2022 May 13;13(5):458. doi: 10.1038/s41419-022-04865-y (PMC9106715; doi:10.1038/s41419-022-04865-y)
Supplement: Supplementary file 7 — Supplementary Table 1 [file 41419_2022_4865_MOESM7_ESM.pdf]

Supplementary Table 1. Mass spe[illegible]

Accession	-10lgP	Coverage (%)	Area	#Peptides	#Unique
Q15717|HuR_HUMAN	283.56	37	3.44E+05	2	2
P63162|RSMN_HUMAN	279.56	17	3.27E+05	1	1
Q92611|EDEM1_HUMAN	270.35	20	2.86E+05	1	1
P62979|RS27A_HUMAN	263.41	31	2.09E+05	3	11
P0CG47|UBB_HUMAN	255.82	25	5.33E+04	8	8
Q9UPW5|CBPC1_HUMAN	248.69	20	2.74E+04	10	10
Q7RTS9|DYM_HUMAN	244.72	10	3.56E+04	3	3
Q9H7B2|RPF2_HUMAN	237.24	5	9.78E+04	2	2
Q9Y6X0|SETBP_HUMAN	232.41	15	1.17E+03	4	6
Q9Y3S1|WNK2_HUMAN	218.72	9	2.47E+03	8	2
O75691|UTP20_HUMAN	215.72	12	1.65E+04	4	14
Q96J92|WNK4_HUMAN	210.72	40	2.41E+04	5	4
Q63HN8|RN213_HUMAN	206.41	18	2.28E+04	4	4
P25705|ATPA_HUMAN	201.86	6	2.15E+04	1	2
P62987|RL40_HUMAN	195.72	22	3.63E+03	4	4
P55265|DSRAD_HUMAN	190.24	20	2.19E+03	2	8
P22087|FBRL_HUMAN	184.49	17	5.56E+02	6	7
P40429|RL13A_HUMAN	178.62	15	1.79E+04	1	7
P07237|PDIA1_HUMAN	175.21	9	1.67E+04	2	12
P31689|DNJA1_HUMAN	169.72	7	6.57E+03	2	4

ctrometry anal

Avg. Mass
591415
266521
145732
57747
72876
31824
72671
115583
21414
72251
57813
12414
45869
49673
38595
29889
18997
82841
29183
80891

ysis of RP1-59D14.5 binding proteins

Description
ELAV-like protein 1 OS=Homo sapiens GN=S=HuR PE=1 SV=1
Small nuclear ribonucleoprotein-associated protein N OS=Homo sapiens GN=SNRPN PE=1 SV=1
ER degradation-enhancing alpha-mannosidase-like protein 1 OS=Homo sapiens GN=EDEM1 PE=1 SV=1
Ubiquitin-40S ribosomal protein S27a OS=Homo sapiens GN=RPS27A PE=1 SV=2
Polyubiquitin-B OS=Homo sapiens GN=UBB PE=1 SV=1
Cytosolic carboxypeptidase 1 OS=Homo sapiens GN=AGTPBP1 PE=1 SV=3
Dymeclin OS=Homo sapiens GN=DYM PE=1 SV=1
Ribosome production factor 2 homolog OS=Homo sapiens GN=RPF2 PE=1 SV=2
SET-binding protein OS=Homo sapiens GN=SETBP1 PE=1 SV=3
Serine/threonine-protein kinase WNK2 OS=Homo sapiens GN=WNK2 PE=1 SV=4
Small subunit processome component 20 homolog OS=Homo sapiens GN=UTP20 PE=1 SV=3
Serine/threonine-protein kinase WNK4 OS=Homo sapiens GN=WNK4 PE=1 SV=1
E3 ubiquitin-protein ligase RNF213 OS=Homo sapiens GN=RNF213 PE=1 SV=3
ATP synthase subunit alpha mitochondrial OS=Homo sapiens GN=ATP5A1 PE=1 SV=1
Ubiquitin-60S ribosomal protein L40 OS=Homo sapiens GN=UBA52 PE=1 SV=2
Double-stranded RNA-specific adenosine deaminase OS=Homo sapiens GN=ADAR PE=1 SV=4
rRNA 2'-O-methyltransferase fibrillarin OS=Homo sapiens GN=FBL PE=1 SV=2
60S ribosomal protein L13a OS=Homo sapiens GN=RPL13A PE=1 SV=2
Protein disulfide-isomerase OS=Homo sapiens GN=P4HB PE=1 SV=3
DnaJ homolog subfamily A member 1 OS=Homo sapiens GN=DNAJA1 PE=1 SV=2
