# Supplementary material for: RP1-59D14.5 triggers autophagy and represses tumorigenesis and progression of prostate cancer via activation of the Hippo signaling pathway
Source: Cell Death Dis. 2022 May 13;13(5):458. doi: 10.1038/s41419-022-04865-y (PMC9106715; doi:10.1038/s41419-022-04865-y)
Supplement: Supplementary file 11 — cddis-author-contribution-form [file 41419_2022_4865_MOESM11_ESM.pdf]

DECLARATION OF CONTRIBUTIONS TO ARTICLE

ADMC

Manuscript Number: CDDIS-21-0943

Journal Name: *Cell Death & Disease* (the 'Journal')

Proposed Title of the Contribution:

RP1-59D14.5 triggers autophagy and represses tumorigenesis and progression of prostate cancer via activation of the Hippo signaling pathway (the 'Contribution)

Author(s):

Bing Zhong, Zexue Zhao, Xi Jiang (the 'Authors')

For all *CDDis* articles, each person named as an author in the published version must be able to show he or she has contributed substantially to the article.

Authorship credit should be based on 1) substantial contributions to conception and design, acquisition of data, or analysis and interpretation of data; 2) drafting the article or revising it critically for important intellectual content; and 3) final approval of the version to be published. Authors should meet conditions 1, 2 and 3.

Any person who cannot be shown to have made a substantial contribution to the article cannot be listed as an author in the final version. The name of any person who is deemed to have made a minor contribution can, however, appear in the Acknowledgments section of the article.

Please complete the table below to indicate the contributions of all named authors to the manuscript.

Author Full Name:	Specification of Contribution to the Manuscript:
Bing Zhong	substantial contributions to conception and design; drafting the article and revising it critically for important intellectual content.
Zexue Zhao, Xi Jiang	acquisition of data, analysis and interpretation of data
All the authors	final approval of the version to be published.

Please complete the table below to indicate the contributions of all named authors to the figures.

Figure 1:

Zexue Zhao, Xi Jiang

Figure 2:

Zexue Zhao, Xi Jiang

Figure 3:

Zexue Zhao, Xi Jiang

Figure 4:

Zexue Zhao, Xi Jiang

Figure 5-7:

Bing Zhong

Figure 8:

Bing Zhong

Signed for and on behalf of the Author(s):	Print Name:	Date:
ZeXue Zhao.	Zexue Zhao	20211119
